# Familial risk for gastric carcinoma: an updated study from Sweden

**DOI:** 10.1038/sj.bjc.6603722

**Published:** 2007-04-03

**Authors:** K Hemminki, J Sundquist, J Ji

**Affiliations:** 1 Center for Family and Community Medicine, Karolinska Institute, 141 83 Huddinge, Sweden; 2 Division of Molecular Genetic Epidemiology, German Cancer Research Center (DKFZ), Im Neuenheimer Feld 580, 69120 Heidelberg, Germany

**Keywords:** gastric cancer, cardia cancer, corpus cancer, familial risk

## Abstract

Reliable data on familial risks are important for clinical counselling and cancer genetics. However, the estimates of familial risk of gastric cancer vary widely. We examined the risk of familial gastric cancer using the updated Swedish Family-Cancer Database with 5358 patients among offspring and 36 486 patients among parents. There were 133 families with one parent and one offspring diagnosed with gastric cancer, and 20 families with two affected offspring. Familial standardised incidence ratios (SIRs) were 1.63 and 2.93 when parents and siblings presented with gastric cancer, respectively. The high sibling risk was owing to cancer in the corpus (SIR 7.28). The SIR for cardia cancer was 1.54 when parents were diagnosed with any gastric cancer. Cardia cancer associated with oesophageal cancer, particularly with oesophageal adenocarcinoma. Among specific histologies, signet ring cancer showed an increase. A few associations were noted for discordant sites, including the urinary bladder and the endometrium. *H. pylori* infection, although not measured in the present study, is probably an important risk factor for the high sibling risk of corpus cancer. Familial clustering of cardia cancer is independent of *H. pylori* infection, and may have a genetic basis. The familial association of cardia cancer with oesophageal adenocarcinoma may provide aetiological clues.

Although the incidence of gastric cancer has declined in the past several decades, it remains the second most common cause of cancer-related deaths in the world (Parkin *et al*, 2001). It has been estimated that there were more than 870 000 deaths from this disease in the year 2000 (Pisani *et al*, 1993). In Sweden, gastric cancer amounts to 2.3% of all malignancies (Center for Epidemiology, 2002). Approximately 90% of gastric cancers are adenocarcinomas, whereas leiomyosarcomas, carcinoids and undifferentiated tumours are rare, making up most of the remaining 10%. According to the Lauren classification, adenocarcinomas can be subdivided into two histological types, the intestinal type, with a greatly declined incidence rate, and the diffuse type, exhibiting a relatively constant rate. Most gastric carcinomas are located distal to the cardia (corpus cancer), which has markedly declined in the Western countries, including Sweden; about 25% are localised proximally in the cardia (cardia cancer), with a relatively stable incidence (Desoubeaux *et al*, 1999; Ekstrom *et al*, 1999; Ekstrom *et al*, 2000).

*Helicobacter pylori* is a main risk factor of distal gastric cancer (Huang *et al*, 1998; Crew and Neugut, 2004). The infection causes a chronic active inflammation in the gastric mucosa and, in a subgroup, gastritis arises in the corpus, which is a risk factor for gastric cancer (Uemura *et al*, 2001). Other risk factors of distal gastric cancer include dietary nitrite, salt, and smoking (Hirohata and Kono, 1997; Ye *et al*, 1999; Kobayashi *et al*, 2002; Ngoan and Yoshimura, 2003; Crew and Neugut, 2004). As for cardia cancer, *H. pylori* infection appears not to be important and there may even be an inverse association (Crew and Neugut, 2004; Ye *et al*, 2004). Smoking, dietary nitrite and obesity may be risk factors of cardia cancer (Lagergren *et al*, 1999b; Ye *et al*, 1999; Crew and Neugut, 2004). A positive family history is a risk of gastric cancer but the reported risks ranged widely from about 1.3 to 3.0 (La Vecchia *et al*, 1992; Munoz *et al*, 1997; Lissowska *et al*, 1999; Lagergren *et al*, 2000; Hemminki and Jiang, 2002b; Amundadottir *et al*, 2004; Kerber and O'Brien, 2005). Gastric cancer is a manifestation of several cancer syndromes, such as hereditary nonpolyposis colorectal carcinoma (HNPCC, particularly in Japanese families), adenomatous polyposis (FAP) and some other rare syndromes (Lynch and de la Chapelle, 2003).

In view of the varying familial risk estimates for gastric cancer, we examined here the risk using the newest update of the nation-wide Swedish Family-Cancer Database, which covered offspring between birth and age 72 years and their parents. Compared with the earlier study from this Database based on 58 familial cases in the first-degree relatives (Hemminki and Jiang, 2002b), the extended population included 153 familial gastric cancers, allowing a more exact definition of the familial risk and analysis by specific histological types and anatomical sites. A familial risk of cardia cancer, if demonstrated, would be independent of infection by *H. pylori* (Ye *et al*, 2004). Besides, the associations of gastric cancer with other tumours in families were also examined. The special properties of the Database include registered sources of family relationships and medically verified cancer cases with practically complete national coverage. This is the largest study published on familial gastric cancer.

## MATERIALS AND METHODS

Statistics Sweden maintains a ‘Multigeneration Register’ where children, offspring, born in Sweden in 1932 and later are registered with their biological parents and they are organised as families (Hemminki *et al*, 2001a). The latest version of the Multigeneration Register, which has been incorporated in the Database, includes supplementary data from church records on index persons domiciled in Sweden between 1947 and 1961, including information about biological parents, children, siblings, and adoptions. This Register was linked by the individually unique national registration number to the Cancer Registry to create the Swedish Family-Cancer Database (MigMed2), which was updated in 2006 with cancer cases from 1958 to 2004. The data on families and cancers have a complete coverage, barring some groups of deceased offspring, which affected those born in the 1930s and those who died before 1991. Although this small group of offspring with missing links to parents has a negligible effect on the estimate of familial risk (Dong and Hemminki, 2001), we limited the present study to offspring whose parents were known to eliminate the possibility of bias. Cancer registration is currently considered to be close to 100% (Center for Epidemiology, 2002). A four-digit diagnosis code according to the 7th revision of the *International Classification of Diseases* (ICD-7) has been used. The following ICD-7 codes were grouped: ‘upper aero digestive tract’ cancer codes 161 (larynx) and 140–148 (lip, mouth, and pharynx), except for code 142 (salivary glands), ‘non-Hodgkin lymphoma’ codes 200 and 202, and ‘leukaemia’ codes 204–207 (leukaemia), 208 (polycytemia vera) and 209 (myelofibrosis). Rectal cancer, ICD-7 code 154, was subdivided into the anus (squamous cell carcinoma, 154.1) and mucosal rectum (154.0). Basal cell carcinoma of the skin is not registered in the Cancer Registry. Lymphomas are classified as independent entities irrespective of their anatomic sites. Only the first gastric cancer was considered in the present study, and a total of 5358 offspring and 36 486 parents were diagnosed with gastric cancer in the Database. Most Swedish counties recorded gastric cancer with histopathological data by the Systematised Nomenclature of Medicine (SNOMED, http://snomed.org) from year 1990, but the nation-wide application of SNOMED code was available after 1993. To avoid loss of cases, we included those cases diagnosed between years 1990 and 2004 for the analysis of familial risks by histology.

Family history of gastric cancer was determined for each offspring in the beginning of the follow-up period. Gastric cancer incidence was compared among offspring with or without family history. If not specified otherwise, the follow-up was started for each offspring at birth, immigration or on 1 January 1961, whichever was the latest time. Follow-up was terminated at the diagnosis of first cancer, death, emigration or the closing date of the study on 31, December 2004. Standardised incidence ratios (SIRs) were used to measure the cancer risks for offspring according to the occurrence of cancers in their families. SIRs were calculated and results are shown for offspring gastric cancer when the parent, sibling or parent and sibling had any cancer, that is using parents, siblings or parents and siblings as probands in mutually exclusive categories. When more than two affected offspring were found in any family, they were counted as independent events.

Parents' ages were not limited, but offspring were 0–72 years of age. SIRs were calculated as the ratio of observed (O) to expected (E) number of cases. The expected numbers were calculated from 5-year-age, gender, tumour type, period, socioeconomic status (six groups), and residential area (three groups) specific standard incidence rates for all offspring lacking a family history (Esteve *et al*, 1994). Confidence intervals (95 and 99% CI) were calculated assuming a Poisson distribution, and they were rounded to the nearest two decimals (Esteve *et al*, 1994). Risks for siblings were calculated using the cohort method, as described elsewhere (Hemminki *et al*, 2001b).

## RESULTS

### Familial gastric cancer

The Swedish Family-Cancer Database, which covered years 1961–2004 from the Swedish Cancer Registry, included 5358 offspring (0–72 years) and 36 486 parents with gastric cancer. Familial risks for offspring gastric cancer were calculated by using parents, siblings or parents, and siblings as probands, and by adjusting for age, period, residential area, and socio-economic status (Table 1). Only those cancer sites with at least five offspring gastric cancers (column ‘parent only’) were listed in Table 1. Offspring gastric cancers were significantly increased when a parent (SIR 1.63) or a sibling (2.93) was diagnosed with gastric cancer; the risks were significant even at a 1% level, as indicated by underlined SIRs. The associations were mainly related to gastric adenocarcinoma. In order to compare risks for offspring of affected parents and for siblings, we limited the parental ages to 72 years, and the SIR was still lower, 1.64 (*N*=75, 95% CI 1.29–2.06). The risks were increased to 1.76 (*N*=34, 95% CI 1.22–2.46) for offspring of affected parents and to 3.99 (*N*=6, 95% CI 1.43–8.73) for siblings if the subjects were diagnosed at ages younger than 50 years. Conversely, the SIRs were decreased to 1.58 (*N*=99, 95% CI 1.29–1.93) and 2.63 (*N*=14, 95% CI 1.43–4.43) if diagnostic ages were 50 years and higher. We further analysed the risk for those diagnosed after year 1991; the SIRs were 1.79 (*N*=126, 95% CI 1.49–2.13) and 2.89 (*N*=17, 95% CI 1.68–4.63) in offspring of affected parents and siblings, respectively.

Offspring gastric cancer was associated with paternal genital cancers (2.76). Among siblings, gastric cancer was associated with endometrial (1.65) and urinary bladder cancers (1.67). The risk was of borderline significance for colorectal cancers (1.37, 95% CI 0.99–1.87) among siblings. When a parent and a sibling were affected with lung cancer, the SIR for offspring gastric cancer was significantly increased to 4.19. In the 21 families with gastric and endometrial cancers among siblings, three siblings were diagnosed with an endometrial tumour before age 45 years; for parents in these families, three pancreatic, two gastric, two prostate cancers, and one colon cancer (age=40) were observed. For the 24 families with gastric and urinary bladder cancers among siblings, three parents were noted with gastric cancer, two parents with pancreatic cancer, and one parent each with liver, lung, breast, and prostate cancers, respectively.

### Familial cardia and corpus cancers

Because of the different risk factors for cardia and corpus cancers, we further analyzed the familial risks for the two anatomical sites based on SNOMED. For cardia cancer (Table 2), significantly increased SIRs were observed when parents were diagnosed with all oesophageal cancer (2.94), oesophageal adenocarcinoma (5.67), and gastric cancer (1.54). When cardia cancer was identified by the ICD-7 code and diagnosed after age 49 years (covering years 1970–2004, data not shown), a significant association was noted for nervous system tumours (SIR=2.42, *N*=9, 95% CI 1.10–4.62) among siblings. Nervous system tumours were further analysed by histology; the SIR was 4.48 (*N*=5, 95% CI 1.41–10.55) and 3.36 (*N*=5, 95% CI 1.06–7.91) when siblings presented with glioblastoma and any glioma, respectively.

The risk for offspring corpus cancer was significantly increased to 2.50 and 3.70 when parents were diagnosed with gastric and corpus cancers, respectively (Table 2). The risk was high when siblings were presented with gastric cancer (7.28). For discordant cancer sites, offspring corpus cancer was associated with maternal breast cancer (1.84) and non-Hodgkin's lymphoma among sibling (4.77). An association was also noted for maternal ovarian cancer (2.02, *N*=17, 95% CI 1.17–3.24) when corpus cancer was recorded by ICD-7 (data not shown).

### Familial gastric cancer by histology

We further analyzed the risks for histology specific gastric cancer in offspring by familial gastric cancer (Table 3). Offspring unspecified adenocarcinomas showed significantly increased risks when parents (1.79) or siblings (2.90) were diagnosed with gastric cancer. The risks for signet ring cancer were increased when parents (1.99) or siblings (5.40) presented with gastric cancer. Five of these signet ring cancers were located in the corpus and two in cardia; for the others the location was unspecified.

## DISCUSSION

The sharp decline in gastric cancer incidence during the past decades suggests that environmental risk factors play an important role in its aetiology. A Nordic twin study estimated that 62% variation in gastric cancer were owing to random environmental effects, 10% from shared environmental factors, and 28% from heritable effects (Lichtenstein *et al*, 2000); however, only the first figure was significant. Genetic and environmental components for gastric cancer have also been estimated based on the Swedish Family-Cancer Database (Czene *et al*, 2002). The results agreed with the twin study on the importance of random environmental effects (71%). Both adult (15%) and childhood (13%) shared environmental effects were also significant. Only 1% was assigned for heritable effects. Another piece of evidence on the environmental aetiology of gastric cancer has been the concordance of gastric cancer between spouses (Hemminki and Jiang, 2002a; Amundadottir *et al*, 2004).

In the present study 35 cancer sites, three groups of probands, different age groups, and histological types were covered and some associations were likely to appear by chance. However, only one SIR was significantly decreased, compared with 10 significantly increased ones (Table 1), suggesting that chance findings could be limited. We also calculated the 99% CI to deal with this issue; true findings could be more possible if they were significant at 1% level. Additionally, significant associations were examined for biological plausibility and consistency with other literatures, as discussed later. Another minor limitation of this study may be the lack of parental links of a few offspring born in the 1930s, who died before 1991. Such families were excluded from the present study as only offspring with known parents were considered. Whether this caused a bias, we calculated the familial risk for offspring diagnosed after year 1991. The SIRs were essentially unchanged, suggesting that the present risk estimates were unbiased. Additionally, data on possible confounding factors, such as diet, *H. pylori* infection, and smoking, were unavailable in the Database: However, the adjustment for socioeconomic status could partly decrease their effects. Moreover, the low association between gastric and lung cancer (1.10, Table 1, parental probands) suggested that smoking was not a confounder of the familial associations.

In the present study we provided evidence for familial risk of gastric cancer in first-degree relatives, which may be caused by heritable and/or shared environmental effects. Significant associations were observed for offspring of affected parents with a SIR of 1.63 and for siblings with a SIR of 2.93. However, sibling risks were not fully comparable with offspring risks in this study, because of the different age distribution for the offspring and parental generations in our Database. To compare the risks for offspring and siblings, the maximal parental age was limited to 72 years; however the SIR for offspring (1.64) was unaltered. The difference between sibling (3.99) and offspring risks (1.76) was even higher when the age of onset was limited to lower than 50 years. The high sibling risks may be owing to shared childhood environmental factors or to a recessive mode of inheritance, or both. A recent study by Chang *et al* (2002) showed that the prevalence of *H. pylori* infection was 89% when a sibling had a history of gastric cancer, giving an OR was 5.3; this was higher than the OR of 2.1 when a parent presented with gastric cancer. Thus subjects who had a sibling with gastric cancer had a higher risk of *H. pylori* infection than subjects with a parental history of gastric cancer.

*H. pylori* infections are still common in Sweden. A sample of 1000 Swedes were randomly selected for oesophago-gastro-duodenoscopy with biopsies and *H. pylori* serology and one-third of the subjects had a current infection and a further 10% had signs of past infection (Storskrubb *et al*, 2005). The prevalence of premalignant atrophy and intestinal metaplasia of the corpus was high among those with current and past infection while seronegativity excluded such precancerous conditions in the corpus. Many lines of evidence suggest that *H. pylori* infection is related to corpus cancer, rather than to cardia cancer (Siman *et al*, 1997; Eslick *et al*, 1999; Kelley and Duggan, 2003; Ye *et al*, 2004). Familial risk of corpus cancer was significantly increased when parents presented with any gastric cancer (2.50). The SIR was even high for concordant corpus cancers (3.70), suggesting that parents with corpus cancer were more likely carriers of *H. pylori* infection. The risk for corpus cancer among sibling was very high (7.28), in agreement with the evidence that cross-transmission of *H. pylori* infection was more common between siblings than between parents and offspring (La Vecchia *et al*, 1995; Goodman and Correa, 2000). The consistency with the transmission patterns of *H. pylori* infection, the high sibling risks, particularly for concordant corpus cancers, suggest that *H. pylori* infection was an important risk factor for the familial risk of corpus cancer in the present study.

Evidence was provided for an independent familial risk for cardia cancer in the offspring of affected parents diagnosed with gastric cancer (1.54). An earlier Swedish case–control study showed a moderate increase of cardia cancer among subjects with a familial history of gastric cancer in first-degree relatives. However, according to the authors, the result could be overestimated by reporting bias (Lagergren *et al*, 2000). A non-significant excess of cardia cancer was also noted in an US study among individuals reporting a family history of digestive tract cancers (Dhillon *et al*, 2001). In the present study, we observed a strong familial association between oesophageal adenocarcinoma and cardia cancer, giving an SIR of 5.67. Infection with *H. pylori* could not explain the observed associations, because neither cardia cancer nor oesophageal adenocarcinoma are related to *H. pylori* (Ye *et al*, 2004). Barrett's oesophagus is an established risk factor of both oesophageal adenocarcinoma (OR 43.5) and gastric cardia cancer (OR 4.4) (Lagergren *et al*, 1999a). Familial clustering of Barrett's oesophagus has been described (Crabb *et al*, 1985; Jochem *et al*, 1992), suggesting that the hereditary components for Barrett's oesophagus could be related to both cardia and oesophageal adenocarcinomas. Other factors, either genetic or environmental, may be also related to the family aggregations of cardia cancer (Chow *et al*, 1998; Zhang *et al*, 2006).

The histological classification used by the Swedish Cancer Registry does not specify intestinal and diffuse types of the Lauren classification and we were thus unable to contribute to the existing literature (Lehtola, 1978; Palli *et al*, 1994; Lissowska *et al*, 1999). Germ line mutations in the E-cadherin gene have been reported in Maori kindred with hereditary diffuse type gastric cancer (Guilford *et al*, 1998). In the present study, signet ring cancer resembles diffuse type carcinoma (Hamilton and Aaltonen, 2000), which showed a significant increase when parents or siblings presented with gastric cancer.

Gastric cancer is a component in several cancer syndromes, of which HNPCC, a dominant disease, is the most common (Lynch and de la Chapelle, 2003; Zhang *et al*, 2006). The most common cancer site in HNPCC is the colorectum, which showed a borderline increase among siblings in the present study. Other HNPCC-related cancers, endometrial and urinary bladder cancers, and brain gliomas were in excess but only among siblings, leaving the association to HNPCC in doubt.

The associations of gastric cancer with lung and bladder cancers could be related to tobacco smoking, because of the known familial aggregation of the smoking habit (Fisher, 1958; Tokuhata and Lilienfeld, 1963; Jonsson *et al*, 2004; Madden *et al*, 2004; Lorenzo Bermejo and Hemminki, 2005). Gastric and lung cancers could also be linked by *H. pylori* infection (Gocyk *et al*, 2000). However, corpus cancer showed no association with lung cancer. Male genital cancers associated with all gastric cancer, but the significance of this observation remains to be established. Human papilloma virus is a risk factor for male and female genital cancers (zur Hausen, 1999), and an association of gastric cancer with female genital cancers has been reported earlier (Goldgar *et al*, 1994). Corpus cancer associated with non-Hodgkin's lymphoma. *H. pylori* infection is an established cause of gastric lymphomas of mucosa-associated lymphoid tissue (Wotherspoon *et al*, 1991; Parsonnet *et al*, 1994), but we had no details of the non-Hodgkin's lymphoma subtypes. The association of corpus cancer with breast cancer was in line with a US case–control study (Dhillon *et al*, 2001). Second corpus cancer has been observed to be in excess after primary sporadic and familial breast cancers (Ji and Hemminki, 2006); BRCA1, BRCA2, and E-cadherin gene may be shared genetic risk factors for the two cancer sites.

In conclusion, the present study showed that familial risks for concordant gastric cancers were higher for siblings than for parents and offspring. The excess sibling risk was mainly owing to corpus cancer for which *H. pylori* infection may be an important contributing factor; however, we have no data on *H. pylori* infection. Familial clustering for cardia cancer could be independent of *H. pylori* infection, as well as the association of cardia cancer with oesophageal adenocarcinoma. The associations with some discordant sites were probably explained by HNPCC and familial aggregation of smoking but all the discordant associations need to be confirmed.

## Figures and Tables

**Table 1:** Risks for gastric cancer in offspring by familial cancers (1961–2004)

	**Parently only**				**Sibling only**				**Parent and sibling**			
**Familial cancer sites**	**O**	**SIR**	**95%CI**		**O**	**SIR**	**95%CI**		**O**	**SIR**	**95%CI**	
Upper aerodigestive tract	37	1.09	0.77	1.51	10	1.14	0.54	2.11	0			
Oesophagus	14	1.14	0.62	1.92	5	1.95	0.61	4.58	0			
Stomach	133	**1.63**	1.36	1.93	20	**2.93**	1.79	4.53	2	4.93	0.46	18.13
Adenocarcinoma	116	**1.63**	1.34	1.95	18	**2.90**	1.71	4.59	2	6.02	0.57	22.13
Colorectum	182	1.04	0.90	1.21	41	1.37	0.99	1.87	4	1.17	0.30	3.03
Liver	55	1.12	0.85	1.46	4	0.70	0.18	1.82	0			
Pancreas	49	1.03	0.76	1.36	9	1.41	0.64	2.69	0			
Lung	112	1.10	0.90	1.32	32	1.34	0.92	1.90	6	**4.19**	1.51	9.19
Breast	150	1.04	0.88	1.22	84	1.10	0.88	1.36	3	0.49	0.09	1.45
Cervix	31	1.06	0.72	1.50	12	1.17	0.60	2.04	0			
Endometrium	41	1.08	0.77	1.47	21	**1.65**	1.02	2.53	1	2.92	0.00	16.75
Ovary	42	1.25	0.90	1.69	20	1.58	0.97	2.45	0			
Other female genital	8	1.12	0.48	2.21	0				0			
Prostate	207	1.00	0.87	1.15	40	1.11	0.79	1.51	5	0.83	0.26	1.96
Other male genital	7	**2.76**	1.09	5.72	2	2.11	0.20	7.75	0			
Kidney	50	1.04	0.77	1.37	10	1.01	0.48	1.86	0			
Urinay bladder	71	1.03	0.80	1.30	24	**1.67**	1.07	2.49	3	4.98	0.94	14.73
Melanoma	25	0.90	0.58	1.33	26	1.18	0.77	1.74	0			
Skin	51	0.91	0.67	1.19	11	1.38	0.69	2.48	0			
Nervous system	33	0.92	0.63	1.29	25	1.32	0.85	1.95	0			
Thyroid gland	10	0.96	0.46	1.78	9	1.55	0.70	2.96	0			
Endocrine glands	12	0.55	0.28	0.97	16	1.55	0.88	2.52	0			
Connective tissue	13	1.45	0.77	2.49	6	1.87	0.67	4.10	0			
Non-Hodgkin's lymphoma	49	1.25	0.92	1.65	19	1.34	0.80	2.09	0			
Hodgkin's disease	8	1.23	0.53	2.44	2	0.63	0.06	2.30	0			
Myeloma	24	1.02	0.65	1.51	2	0.50	0.05	1.83	0			
Leukemia	35	0.85	0.59	1.18	10	1.03	0.49	1.90	0			
All	1104	1.04	0.98	1.10	231	**1.18**	1.04	1.35	205	**1.26**	1.09	1.44

CI=confidence interval, O, observed cases. Bold type: 95%CI does not include 1.00; underline type, 99%CI does not include 1.00.

**Table 2:** Risks for cardia and corpus cancers in offspring by familial cancers (1993–2004)

	**Parently only**				**Sibling only**			
**Cancer sites**	**O**	**SIR**	**95%CI**		**O**	**SIR**	**95%CI**	
*Risk for cardia cancer*								
Oesophagus	8	**2.94**	1.26	5.82	1	1.83	0.00	10.52
Adenocarcinoma	3	**5.67**	1.07	16.80	0			
SCC	4	2.17	0.57	5.62	0			
Stomach	28	**1.54**	1.02	2.23	1	0.67	0.00	3.83
Cardia	0				0			
Corpus	11	1.20	0.60	2.15	1	2.33	0.00	13.33
								
*Risk for corpus cancer*								
Oesophagus	0				1	6.27	0.00	35.94
Stomach	12	**2.50**	1.29	4.38	3	**7.28**	1.37	21.55
Cardia	0				0			
Corpus	9	**3.70**	1.68	7.06	1	2.33	0.00	13.33
Breast	15	**1.84**	1.02	3.04	4	0.89	0.23	2.31
Non-Hodgkin's lymphoma	4	1.80	0.47	4.66	4	**4.77**	1.24	12.34

CI=confidence interval, O, observed cases. Bold type: 95%CI does not include 1.00; underline type, 99%CI does not include 1.00.

**Table 3:** Risks for offspring histology specific gastric cancer by familial gastric cancers, (1990–2004)

**Histologies**	**O**	**SIR**	**95%CI**		**O**	**SIR**	**95%CI**	
Stomach, all	128	**1.76**	1.47	2.09	17	**2.80**	1.63	4.49
Adenocarcinoma	81	**1.79**	1.42	2.23	11	**2.90**	1.44	5.20
Carcinoid	5	2.26	0.71	5.32	0			
Signet ring cancer	18	**1.99**	1.18	3.15	4	**5.40**	1.40	13.95
Gastrointestinal stromal tumours	3	1.23	0.23	3.63	1	5.34	0.00	30.60

CI=confidence interval, O, observed cases. Bold type: 95%CI does not include 1.00; underline type, 99%CI does not include 1.00.
